# Notes from My Case-Book

**Published:** 1852-10

**Authors:** Wm. H. McKee

**Affiliations:** Raleigh, North Carolina


*Notes from my Case-Book.* By Wm. H. McKee, M. D., of Raleigh, North Carolina.

Messrs. Editors,—I offer the following cases from my note-book, and as occasion admits will present such others as I may think likely to be of interest, and repay the trouble of perusal.

### *Prolapsed Bladder Obstructing Labor.*

The subject of the present case was a colored woman, in labor with her third child, of robust and strong constitution. She was taken in labor on Wednesday night, at which time a colored midwife was sent for. She flattered the woman that the labor would soon be over, and all was right. She continued to linger, and suffered greatly, from her account, with cramp. The membranes gave way on Thursday afternoon, at which time she enjoyed a few hours of refreshing sleep. The pains continued, alternating occasionally with intermission of ease; finally, the midwife becoming alarmed, she desired that a physician should be sent for, and on Friday afternoon a very intelligent neighborhood physician, Dr. J. B. Owen, was called in. At the time of his arrival, he found the pains as he thought insufficient, and was about to administer a dose of ergot, but before doing so he made an examination, per vaginam, and found that there was an obstruction to the labor, which was unusual, and requested that I should be sent for. He then gave her an anodyne to procure rest. Accordingly, I was called in on Saturday; the distance was about fourteen miles, and I did not see the patient until one o'clock in the afternoon, she having been in labor some seventy hours. The following was her condition:—pulse 120 and feeble; countenance anxious, and wore a peculiar expression, an aspect of anxiety mixed with distress, and complained of pain in the hypogastric region, with a dragging sensation from the umbilicus, as if she would burst at each pain; skin bathed in a cold and profuse perspiration; tongue red and dry; thirst great; pains strong and forcing; external organs of generation swollen as thick as the wrist, hard, and at each pain blood exuded from the labiæ, they having become very sore, cracked, and dry from the repeated touching of the midwife. On examination, per vaginam, my finger came in contact with a large elastic tumor, filling up the vagina, so I found it impossible to reach the presenting part of the fœtus. My first impression was, that it was the membranes, being assured that they had given way on Thursday. I inquired the last time she had micturated, and learned that it was about twenty-four hours. With these facts of the case, I pronounced it a case of prolapsed bladder. I first attempted to introduce the catheter, but failed, I am confident, for the want of a proper instrument, and from the frequent appeals of the patient for relief, I introduced a thumb lancet guided by the index finger into the vagina, and tapped the bladder, when immediately followed a large quantity of urine, to the great relief of the patient, and in fifteen minutes thereafter, she gave birth to a large dead male child. The after treatment, which was conducted by Dr. Owen, consisted in anodynes, with cooling drinks, cleanliness and an occasional aperient. She recovered in the usual time without having had a bad symptom, and resumed her ordinary business as a field hand.

*Remarks.*—This is the only case of prolapsed bladder which I have met with in a practice of twelve years. Ramsbotham, in his late work on obstetrics, mentions it as a cause of lingering labor, and condemns tapping the bladder in such cases; but it was impossible to have done otherwise in the present case, and the result proved it was judicious. I cannot conceive why the bladder should not be tapped when prolapsed in labor, as well as in cases of retention from stricture in the uretha, stone in the bladder, or enlarged prostrate in the male. It seems that the risk would be much less, but, I readily admit, that if the catheter can be used, it should by all means be preferred, and all operations to be avoided if possible.

### *Retention of the Placenta.*

In volume iv. page 177, of the New Orleans Medical Journal, will be found an article, by Dr. E. D. Fenner, upon this subject. Since reading that article, I have had two such cases to occur in my practice. The first was that of a lady with her ninth child. She had given birth to four living children, and had had four abortions. In the present case, she was about six months gone, and after unusual exertions and fatigue upon her part, in arranging her house for the summer, she was taken with pain in the lumbar region, which was followed by a slight hemorrhage. Thinking but little about it, she continued to pursue her ordinary business for nearly a fortnight, during which time there was more or less hemorrhage, until her strength became very much enfeebled, and she took her bed. I was then consulted about the case, and gave as my opinion that the fœtus must be in a decaying state, and that the sooner she lost it the better. The pains and flooding both continued to increase; an anodyne of morphia, with a mustard plaster to the back, was directed. This procured a good night's rest, and on the next morning having a call to pass her urine, and while in the attempt to do so, she was seized with pain, and immediately passed a decayed fœtus into the night glass, separating itself at the same time from the placenta, the funis being in a sphacelated state. Being sent for immediately, I was in the house in half an hour after it happened; found her free from pain, no hemorrhage, but very uneasy about the after birth. On making an examination, per vaginam, I found the mouth of the womb nearly closed, so much so, that it was with difficulty that I could introduce my finger; to extract it I found was impossible. At her solicitation, I administered thirty grains of ergot, but previous to doing so, I told her and her husband that it was my conviction that it would do her no good, and might be of some injury; but she said that she had taken it before with advantage, and having such a horror of the after birth being retained, she was willing to submit to any means, within reason, to get rid of it. Accordingly the ergot was administered, and was followed by vomiting and great prostration. Brandy, warmth to the extremities, and sinapisms to the stomach were all used, yet no uterine pains followed. I then gave her a full anodyne, and explained to her the situation of the case, and related to her similar ones. By this means her fears became quieted, and she rested well during the day and all that night. On the next morning a dose of castor oil was administered, which operated well, but no placenta followed. A putrid and very offensive discharge from the vagina had now began, for which a diluted solution of chloride of soda was used by injection. This state of things continued for eleven days. During this time she had but very little hemorrhage or pain, and no fever. A dose of morphia, and an occasional dose of oil were administered. At the expiration of this time, she discharged the placenta in a semi-putrid condition. From then she began to recover, and mended rapidly, and since has given birth to two living children.

The second case I did not see until the third day after her confinement. She was at that time suffering a good deal of pain, with more or less hemorrhage. On making an examination, per vaginam, I found that I could not succeed in introducing my hand or finger into the womb, she having become so very sore from the repeated manipulations of the midwife. A dose of laudanum was directed, and in the course of the day a dose of oil. The oil operated very well, and a goodly number of large clots accompanied the operation, and the patient and her attendants thought that she had wasted the placenta, and so informed me on my next visit; but on further inquiry, and another examination, I found that she was mistaken. The usual course, as was in the other case, was enjoined and pursued. After the seventh day, in the afternoon, having taken a dose of oil and spts. turpentine, the placenta came away without any pain, and she recovered in a short time, without ever having had fever or a bad symptom.

*Rupture of the Uterus.*

This case was a colored woman, who was in labor with her twelfth child. All of her previous labors, with the exception of the eleventh, were natural, and attended with no difficulty; but with the eleventh, she suffered very much, and came near dying. In the present labor a very intelligent mid-wife was called in. Labor continued active for three days, during which time she was bled and purged by oil. On the night of the third day the pains became very violent; she suffered from sick stomach, and, after a violent paroxysm of pain, she immediately expressed herself as though the child had moved, and that she was perfectly easy. The mid-wife having exhausted all her means, as she said, to bring on the labor again, and failing, she requested that a physician might be sent for. It was sixteen miles distant from town, and the pains having ceased on Sunday night, it was Monday afternoon before they made application for assistance. I was sent for, but could not go. It being a busy time with the physicians, they were unable to obtain the personal services of a single one. On applying to Dr. I. J. Haywood, the oldest and most experienced physician in the city, and representing the situation of the case, the Doctor told them that he would send some ergot, but if it failed to produce pain, that they must let him know it. The ergot was administered, and without the desired effect. The messenger returned again that night, and reported the result. Dr. Haywood was unable to go to see the patient, as he had told them in the first instance, but feeling that natural desire to mitigate human suffering whenever in his power, sent for his brother, Dr. E. Bush Haywood, who had just graduated and returned home from Philadelphia, and requested that he would go and see her. Though late at night, dark, and a long distance over a bad road, the Doctor without hesitation obeyed the summons promptly.

On arriving at the place, Tuesday morning about day, he found the woman free from pain, but very much swollen. Upon examination, per vaginam, he was unable to reach the mouth of the womb, but after some exploration he did so, and was unable to discriminate what was the real situation or position of the fœtus. He gave her more ergot without producing any pain, and then gave as his opinion that nothing could be done that would save the woman's life; that she was in a sinking condition, and then left for the city, having remained until Tuesday afternoon. The Doctor, feeling a deep solicitude for the case, applied to me to accompany him to see the patient, as his brother's business was such that he could not spare the time. I did so with pleasure on Wednesday morning. Finding, on our arrival, that she had died the previous night, a request was made of her master to let us make a post-mortem examination. He consented, whereupon we proceeded to make it. The weather was very warm, and mortification having taken place, it was with great difficulty that we could accomplish our undertaking. The body was very much swollen, tympanitic, and, on introducing the scalpel into the abdomen, a stream of offensive gas issued, so putrid as to force us from the room for a short time. Having no chlorine, we oiled our hands, and by the burning of tar, we prosecuted and executed our task. On making an incision from the scrobiculus cordes down the linea alba to the pubis, and then a conical one across the middle of the abdomen, we were enabled to present at once a full view of the whole situation. The child was found very much compressed, and in a state of decomposition, cross-wise its mother, out of the womb, with its face in the right iliac fossa, and its breach resting in the left; presenting its sternum to the vaginal orifice. The womb having been located in the abdominal side, from its fundus to its mouth, and found lying behind the fœtus, resting upon the sacrum. Owing to the gangrenous condition in which we found everything, we were compelled to suspend our observations. The result of the observation gave to the owner of the woman the satisfaction that all hopes for her relief were beyond the skill of any man at the time he sought assistance from the profession, and complimented my young friend, Dr. E. B. Haywood, for his prompt and discriminating judgment, besides thanking him for his kind attention.

*Empyema.*

This case I did not attend in its incipient stage, but was called in some two months afterwards. I found it to be a boy of twelve years of age, badly grown, and had been very much exposed to the winter's weather, as it was in the month of March that I first saw him. He was at that time suffering with acute pain in the right side, extending from the ninth rib up to the fourth: Fever, cough, no expectoration, and was unable to lie down, but had to set in a semi-recumbent position, inclining laterally to the right. Previous to my seeing him, he had been bled, blistered and salivated. His respiration was quick, pulse 120, thirsty, and at night delerious. Percussion flat on the whole of the anterior portion of right side, but on the posterior, resonant. Respiration inaudible anteriorly, but audible and somewhat tubular posteriorly. The left lung appeared to labor much more than was natural. On inspecting the thorax, the right side was rather larger than the left; and on measuring, was some two inches larger, the anterior protruding under the nipple. This part of it appeared as if it was glazed, and rather pulpy or doughy to the touch, but gave no indication of fluctuation. Ordered an anodyne at night, poultice to the side, nourishment, flax-seed lemonade as a drink. With this treatment, and regulating the bowels by injections, the abscess pointed between the sixth and seventh ribs, in about ten days. An incision was made at this point with a small trocar having the canula within, and a large quantity of thick pus was discharged. A continuation of the poultices ; with quinine, and muriated tincture of iron, full diet and some porter, he recovered perfectly in the course of two months.

Case the second occurred in a negro man, aged 26, eight miles from the city. His master gave me the following history of his case. The negro had, as they supposed, an attack of pleurisy, for which he had been bled, puked, purged and blistered, and they thought then they must have cured him, but yet he continued to fall off. Though he ate very heartily, he complained of its giving him pain, and when he was first taken sick, three months previous, that he was as black a negro as is usually found, and now he was almost a mulatto. He suffered from rheumatism in early life, and then had a stiff knee from it. On inspecting the chest, I found nothing very remarkable as to the general appearance, but on percussing it, the left inferior portion was flat and painful anteriorly, but resonant in the upper two-thirds. By making pressure under the false ribs, in the hypochondriac region, it produced cough and gave him pain. I pronounced it an Empyema, and explained his situation to his master, but he thought that it could not be so, and intimated that he must be feigning his sickness, or, as the negro had it, that he was *tricked*, a very common impression among the negro population, and I am sorry to state, that it is one that is a good deal believed by some of the whites also. In about two weeks the negro began to break out in sores all over him. He had an inordinate appetite, but would refrain from eating as long as he could, because whenever he did eat, it gave him so much pain that he could only get ease by laying on his back, with his heels upwards against the house or fence, and made so much noise as to disturb the rest of the family. After the breaking out commenced, he had a difficulty in passing his urine, and his master was then convinced that he had a venereal disease, which the negro protested was not so : and he sent him off into an out house away from the rest of the family, as if some polluted plague had infested his plantation. He sent for me and communicated his convictions. I made an examination, and assured his master that he had no venereal disease, and that the breaking out was caused by the absorption of the matter which was taken into the circulation, as well as the reason for his turning yellow. On examining his chest, I found an enlargement of the left side, but he was so emaciated, hectic, with night sweats, that I advised a palliative course, and stated that in a few days more they would not be disturbed by the negro, and requested that they would let me know when he died, so that I might make a post-mortem. Three days afterwards I was notified of his death. On arriving at the place, a large number of the neighbors had collected to witness the examination. The sternum was removed in the usual manner in making an examination of the chest, when a large quantity of putrid matter made its escape from the left side, so offensive that I could not continue my observations much further. The lung was compressed, and the pleura very much thickened.

Having satisfied myself and the owner, of the correctness of my opinion in the case, I proceeded no further with the examination. What is only remarkable in this case was the gradual fading of the dark color, and that he should have become yellow, as well as the breaking out with sores. The over distension from eating was evidently the cause of pain from that source.

